# Analyzing Public Interest in Metabolic Health-Related Search Terms During COVID-19 Using Google Trends

**DOI:** 10.7759/cureus.15715

**Published:** 2021-06-17

**Authors:** Alec D McCarthy, Daniel McGoldrick

**Affiliations:** 1 Department of Surgery, University of Nebraska Medical Center, Omaha, USA; 2 Department of Computer Science, California State University, Monterey Bay, Seaside, USA

**Keywords:** covid-19, metabolic health, google trends, social data, metabolism, obesity, public interest, coronavirus, sars-cov-2, scientific communication

## Abstract

Background

In late 2019, severe acute respiratory syndrome coronavirus 2 (SARS-CoV-2) emerged as a novel virus and initiated a series of events that culminated in the global coronavirus disease 2019 (COVID-19) pandemic. Throughout 2020 and the first half of 2021, massive investigational efforts towards identifying, treating, preventing, and slowing the spread of COVID-19 were carried out. Several predictors for clinical outcomes relating to metabolic health were identified.

Aim and methods

This study aimed to investigate how public interest in search terms associated with metabolic health has changed throughout and during the course of the COVID-19 pandemic. Google Trends was utilized as a tool to gather and compare public interest data in a variety of search phrases. The relative search values were plotted over time, compared pre-and post-COVID-19, analyzed for correlation, assessed for trend directionality, and checked for trend inclusion.

Results

The public interest measured by relative search volume in “metabolic health,” “exercise,” “home exercise," “health,” and “how to improve fitness” significantly increased from pre- to post-COVID-19 pandemic onset while “diet” and “fitness” significantly decreased. The search terms “COVID” and “coronavirus” made up more than 95% of screen queries incorporating COVID-19. During the COVID-19 pandemic, “diabetes” and “weight loss” had the most significant increases in search volume.

Conclusions

Given the changes in public interest observed throughout the course of the COVID-19 pandemic, it is clear that the association between metabolic health and COVID-19 is being successfully disseminated to the public. However, these changes also warrant increased public education surrounding diet and fitness to align public interest with measures proven to improve the clinical outcomes of COVID-19.

## Introduction

In late 2019, severe acute respiratory syndrome coronavirus 2 (SARS-CoV-2) emerged as a fast-spreading virus that has, to date, infected more than 125 million people and contributed to the death of more than 2.7 million people globally and resulted in the global coronavirus disease 2019 (COVID-19) pandemic [1]. In the United States alone, nearly 30 million people have been infected with more than 540 thousand deaths attributed to COVID-19 [2]. Several public policies were implemented to slow the spread of the virus, including masking mandates, stay-at-home orders, work-from-home policies, and social distancing [3-5]. While evidence shows that these measures have imparted varying degrees of efficacy at slowing the spread of the virus, several key confounding factors and comorbidities in clinical outcomes emerged [6]. The CDC states that the adults with cancer, chronic kidney disease, chronic obstructive pulmonary disease (COPD), down syndrome, heart conditions, immunocompromised states, obesity (BMI > 30 kg/m^2^ but < 40 kg/m^2^), severe obesity (BMI > 40 kg/m^2^), pregnancy, sickle cell disease, smoking, and type 2 diabetes mellitus are at increased risk of severe illness from COVID-19 [7]. Other conditions that might be at an increased risk of severe illness from COVID-19, according to the CDC, include asthma (moderate-to-severe), cerebrovascular disease, cystic fibrosis, hypertension, immunocompromised state, neurologic conditions, liver disease, overweight (BMI > 25 kg/m^2^, but < 30 kg/m^2^), pulmonary fibrosis, thalassemia, and type 2 diabetes mellitus [7]. The majority of the aforementioned contributors to COVID-19 severity are directly or indirectly related to metabolic health. 

The definitions for metabolic health vary, but general definitions include having ideal levels of blood sugar, triglycerides, high-density lipoprotein (HDL) cholesterol, blood pressure, and waist circumferences [8]. Other definitions of metabolic health may be binary (i.e., healthy or unhealthy) and fall into categories including metabolic syndrome, insulin resistance, cardiorespiratory fitness, miscellaneous, or the combination of any of the aforementioned [9]. Relatedly, obese and diabetic patients are at heightened risk of a variety of adverse medical events following infection with SARS-CoV-2 [10]. The hospitalization, in-hospital death, mechanical ventilation, venous thromboembolism, and dialysis are all serious adverse events that are linked to obese and diabetic COVID-19 patients [10-12]. A plethora of cohort, cross-sectional, and meta-analysis studies highlight the profound negative effect obesity, diabetes, and poor metabolic health following has on COVID-19 outcomes [10,12-22]. Notably, type 1 and type 2 diabetes are linked to increased adverse events [23]. Increasing BMI (from overweight to obese) contributed to varying degrees of adverse events, and increased risk for populations in younger age brackets (< 60 years old) as well [19]. The importance of metabolic health in improving clinical outcomes following COVID-19 is substantiated and understood in the medical community. Despite mounting evidence indicating the importance of maintaining good metabolic health, unintentional weight gain is emerging as a complication of the COVID-19 pandemic [24]. To this end, health care workers have called for increased efforts in educating the public on the importance of metabolic health relating to overall health and outcomes following COVID-19 [25,26]. To determine how effectively information regarding metabolic health related to COVID-19 has been disseminated to the public, we analyzed public interest in a variety of metabolic health-related searches using Google Trends (GT) data.

Google Trends is a free tool provided by Google that allows users to compare and map temporal and regional interest in a search phrase [27]. To date, many studies have used GT data to evaluate public interest in elective procedures, predict emerging COVID-19 cases, evaluate symptoms of COVID-19, and explore public interest in COVID-19-related inquiries [28-30] Here, we sought to determine how public interest in search terms specifically related to metabolic health and exercise have changed before and after the start of the pandemic and during the ongoing pandemic. In one scenario, if the public interest in improving metabolic health has increased during the pandemic, we can hypothesize that information regarding the importance of metabolic health as a predictor for COVID-19-related adverse events has been effectively communicated. On the other hand, we hypothesize that GT may highlight areas of public interest that should be targets of increased communicability from the scientific and nutrition communities. In this exploratory analysis, we compared relative search volume (RSV) of a variety of search terms associated with metabolic health over a two-year period. Additionally, we compared RSV in terms with the inclusion of the search term “COVID” to explore changing public interest in each term over the course of the pandemic (from early 2020 until the present).

## Materials and methods

Google Trends data

Google Trends provides two types of data: real-time data, which is a sample covering the last seven days of searches, and non-real-time data, which goes back as far as 2004. Google Trends automatically normalizes search data by dividing each data point by the total searches of the geography and time range, it represents and subsequently compares relative popularity (this controls for population variance by region). The normalized popularity of a search term is scaled from 0-100 based on its proportion to all searches. the searches that have exceptionally low volume (RSV < 1%) are expressed as zero. Additionally, duplicate searches from the same person over a short period of time are filtered from the data set. Notably, in this study, GT data were not case-sensitive (covid and COVID can be used interchangeably with no variance in RSV). Google Trends data are easily downloaded as comma-separated values (CSV), making it a useful tool with readily accessible public interest data by way of relative search volume. 

Data source and accessing

**Figure 1:**
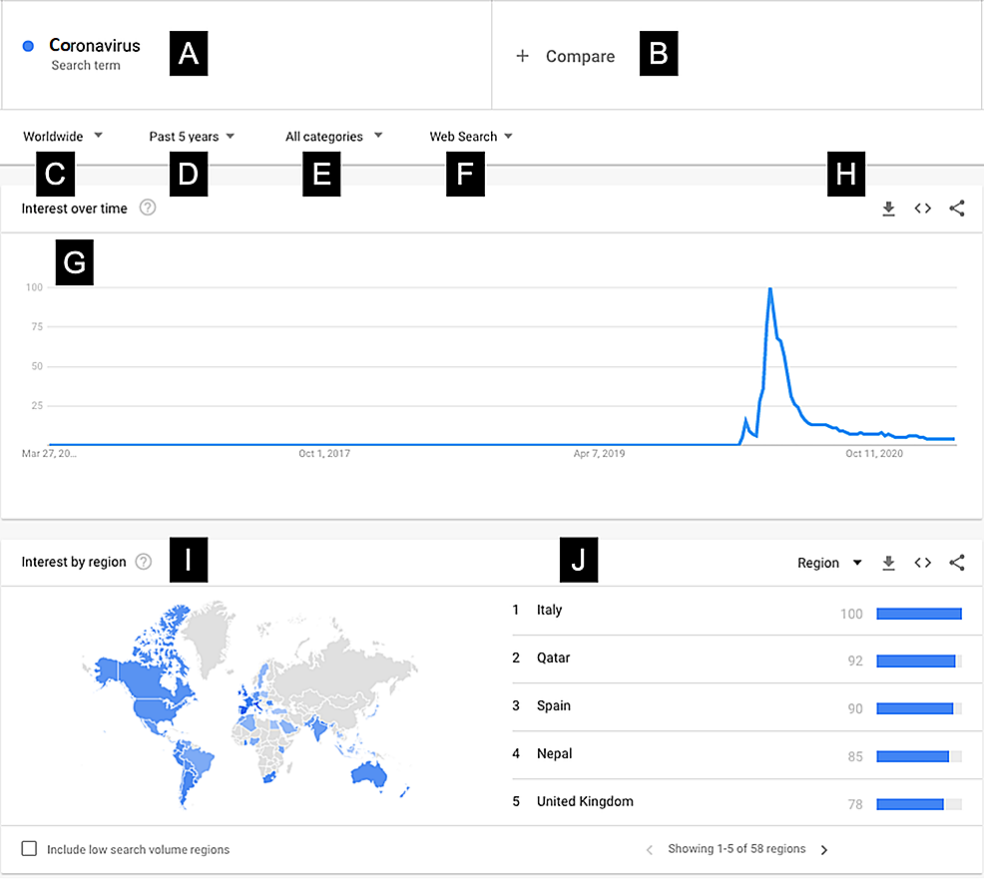
Google Trends interface. Data source: Google Trends (A and B) Phrase search bars. (C) Regional selection criteria. (D) Date selection. (E) Categorical search filter. (F) Search type. (H) Data actionable (download, embed, share from left to right). (I) Interest by region. (J) List of regions with the highest RSV. RSV: relative search volume

All of the data used for this study was gathered using GT. First, a search term (i.e., COVID, coronavirus, obesity, etc.) was selected and searched in the GT search bar with a pre-selected date range and interval. In this study, we measured weekly RSV for each search term between January 6, 2019, and February 28, 2021, to sample a near equal number of weeks pre- and post-COVID-19 (n = 58, n = 53, respectively). Subsequent data sets were downloaded via GT download and stored in a local database for further processing. The search terms were selected based on “related queries” and on researcher discretion. Previous studies have stacked RSV of different terms such that GT expresses search terms relative to one another. An example of the GT user interface with labeled components is given in Figure 1.** **In our study, however, we isolated each term such that the RSV was calculated as a single term’s search frequency over the given time period, relative only to its popularity at different times.

Categorization of search terms

The data generated from GT were categorized into different groups based on a reference point subjectively determined as the first week a sizable uptrend in search queries for “COVID” and “coronavirus” occurred. In this case, data generated before February 23, 2020, was labeled as “pre-COVID” and the data generated after February 23, 2020, was labeled as “post-COVID.” To increase the sensitivity to COVID-related searches, a smaller number of health-related searches were conducted with the addition of “COVID” following the general search term (i.e., instead of “health,” the search query was “health COVID”). The inclusion of “COVID” aimed to increase the situational sensitivity of temporal data by removing spikes in general search terms that may have occurred due to unrelated societal events. 

Statistical analysis

All data were generated using GT searches. Raw data were stored in Google Sheets and analyzed in Graphpad Prism version 9.2 (GraphPad Software, San Diego, CA) or JupyterLab. Several types of analyses were carried out in this study. First, the RSV of each phrase was plotted over a specified time interval, as previously reported [30]. Next, statistical tests were carried out on each search term’s numerical data to analyze the long and short-term variations in the public interest and to identify whether or not changes were stationary, trended, or random. One-way ANOVA was selected for its ability to differentiate between changes in RSV using the sub-samples both before (n = 58) and after (n = 53) the onset of the COVID-19 pandemic, with weekly RSV values used to compute mean values and standard deviation. Pearson’s r was used to analyze the possible associations between search terms and assign an r-value between -1 and 1 (i.e., -1 would represent a perfect negative correlation; 1 would represent a perfect positive correlation). An augmented Dickey-Fuller (ADF) test was used to identify whether the RSV was stationary or non-stationary across both time intervals. ADF values test for the inclusion of a trend over a given time point. The significance in pairwise comparisons was indicated as follows: p < 0.05, p < 0.01, p < 0.001, and p < 0.0001.

## Results

Trends in general search trends 

**Figure 2:**
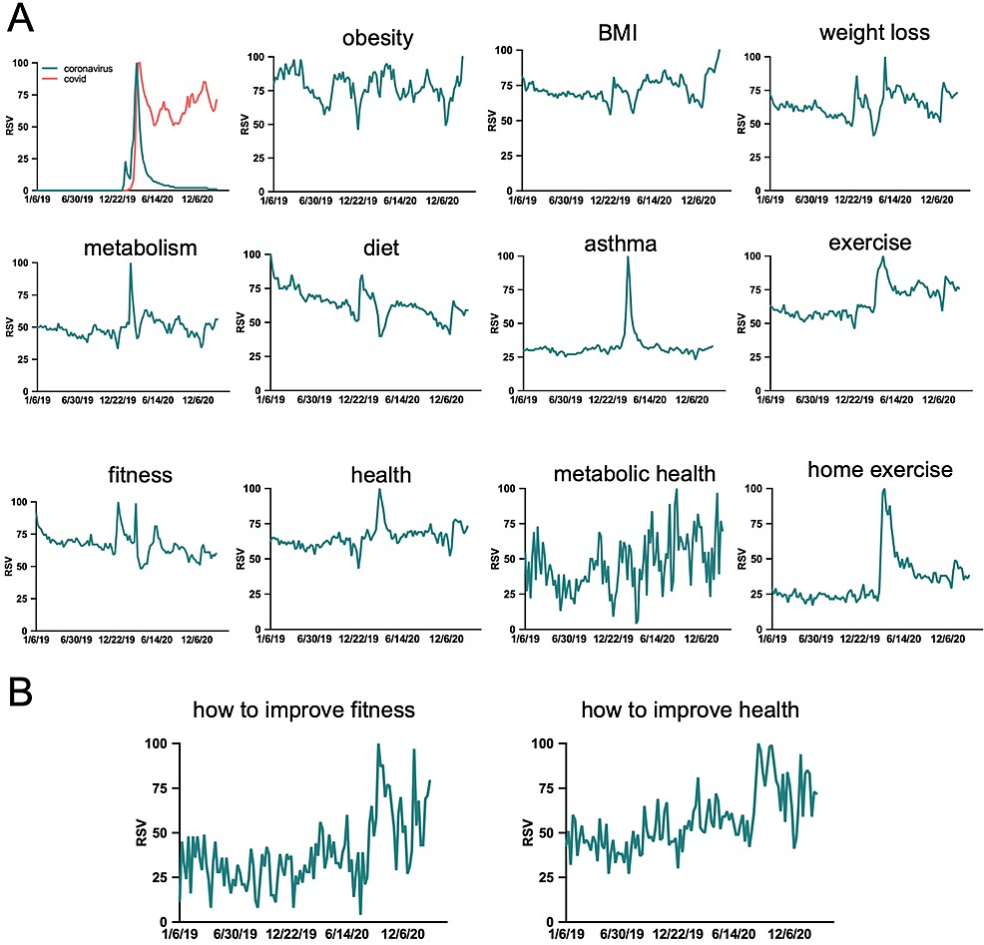
General search terms’ RSV before and after the onset of COVID-19. (A) Relative search volume of words and phrases associated with metabolic health over a two-year period. (B) Relative search volume of specific inquiries in improving health over a two-year period. RSV: relative search volume; COVID-19: coronavirus disease 2019

**Figure 3:**
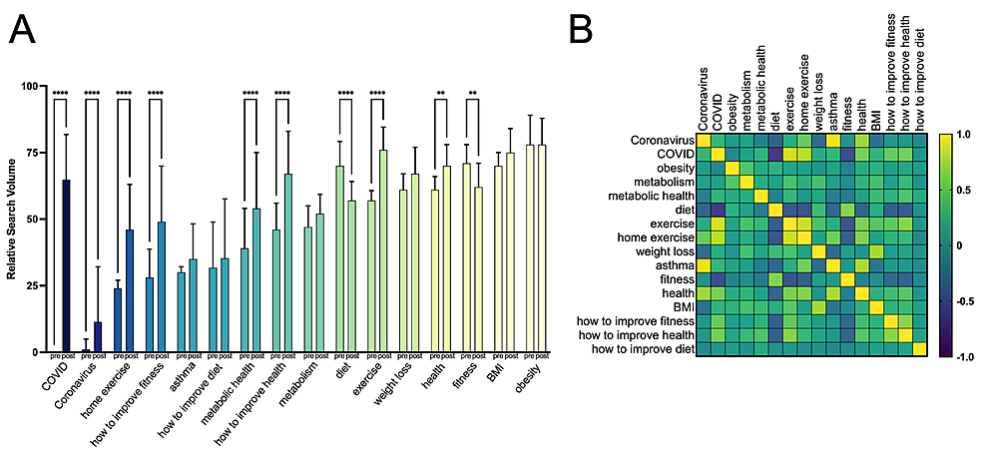
Temporal and associative relationships between metabolic health-related search terms. (A) Weekly average public interest in metabolic health-related searches before and after the onset of COVID-19. (B) Pearson coefficient matrix of metabolic health-related searches over a two-year period. COVID-19: coronavirus disease 2019

An analysis of 13 search terms was carried out to investigate the impact of COVID-19 has had on each term’s public interest over a defined time period (January 6, 2019, to February 28, 2021). RSV data for each term was plotted independently on a scale of 0-100 (Figures 2A, 2B). As a reference point, search terms “COVID” and “coronavirus” were also plotted on the same timeline and achieved the peak RSV on March 22-29, 2020, and March 15, 2020, respectively. Many of the remaining search terms (i.e., fitness, metabolism, asthma, exercise, home exercise, health) saw spikes in RSV that coincided with the initial spike in “COVID” and “coronavirus” searches. Following the onset of the COVID-19 pandemic (subjectively defined here as February 23, 2020), RSV increased most significantly (p < 0.0001) for search terms “metabolic health,” “exercise,” “home exercise,” “BMI,” and “how to improve fitness,” which saw increases of 39.4%, 32.9%, 92.2%, 73.3%, and 44.9%, respectively. The search term “health” saw a significant increase in RSV of 14.3% (p = 0.0003). The search terms “metabolism”(p = 0.2345), “weight loss” (p = 0.0643), “asthma” (0.2345), and “how to improve health” (p = 0.2345) saw insignificant increases of 8.5%, 8.6%, 17.9%, and 7.6%, respectively. Interestingly, three search terms saw decreases in the public interest following the onset of the COVID-19 pandemic. The terms “obesity” (p > 0.9999), “diet” (p < 0.0001), and “fitness” (p < 12.4) saw RSV decreases of -1.08%, -19.14%, and -12.4%, respectively. The average pre- and post-COVID RSV values for each term are summarized and arranged from the smallest pre-COVID RSV to the largest pre-COVID RSV in Figure 3A. All summary data, including date of peak interest, pre and post-COVID RSV, percent change, and adjusted p-values are given in Table 1. 

**Table 1:** General metabolic health-related search terms before and after the COVID-19. RSV: relative search volume; COVID-19: coronavirus disease 2019

Search Term/Phrase	Date of Peak Interest	Pre-COVID RSV	Post-COVID RSV	Change (%)	Adjusted p-Value
Coronavirus	March 15, 2020	1.051	11.389	983.781	<0.0001
COVID	March 22-29, 2020	0.034	64.796	191049.046	<0.0001
Obesity	February 28, 2021	78.458	77.611	-1.079	>0.9999
Metabolism	February 16, 2020	47.475	51.519	8.518	0.2345
Metabolic health	August 30, 2020	38.983	54.333	39.377	<0.0001
Diet	January 6, 2019	70.153	56.722	-19.144	<0.0001
Exercise	April 19, 2020	57.441	76.352	32.923	<0.0001
Home exercise	March 29, 2020	23.695	45.537	92.181	<0.0001
Weight loss	May 3, 2020	61.373	66.667	8.626	0.0643
Asthma	March 15, 2020	30.000	35.370	17.901	0.2345
Fitness	December 29, 2019	70.932	62.130	-12.410	0.0003
Health	March 15, 2020	61.169	69.889	14.254	0.0003
BMI	February 28, 2021	28.305	49.056	73.310	<0.0001
How to improve fitness	August 30, 2020	46.492	67.389	44.949	<0.0001
How to improve health	September 20, 2020	69.678	75.000	7.638	0.2345
How to improve diet	October 25, 2020	31.712	35.33	11.418	0.8358

Correlation between general search phrases

To compare the correlation between search phrases, Pearson’s r was computed and expressed as a matrix heat map with values between -1 and 1, where -1 would represent a perfect negative correlation and 1 would represent a perfectly positive correlation (Figure 3B). Notably, coronavirus + asthma, coronavirus + health, COVID + exercise, COVID + home exercise, BMI + weight loss, asthma + health all had Pearson's r values over 0.7. On the contrary, COVID + diet, COVID + fitness, diet + exercise, diet + home exercise, exercise + fitness, and home exercise + fitness had Pearson's r values of -0.67, -0.54, -0.42, -0.47, -0.48, -0.40, respectively, indicated a negative correlation between the terms.

Sensitivity syntax inclusion

The previous studies using GT data have indicated a potential pitfall as the inability to filter out public interest catalysts that are unrelated to the study. One methodology for increasing sensitivity in GT data is to include a blanket phrase that is highly correlated to the subsequent general search phrases. In this case, we sought to increase the GT data by including a filter phrase in our searches. In order to select the appropriate filter phrase, we analyzed multiple common phrasings (now largely interchangeable) for the COVID-19 pandemic. As noted, GT is not case sensitive, so capitalization for RSV was irrelevant. We analyzed five terms: “COVID,” “SARS-CoV-2,” “coronavirus,” “COVID19,” and “COVID-19.” Brief descriptions of each term are given in Table 2.

**Table 2:** COVID-related syntax for improved search sensitivity. SARS-CoV-2: severe acute respiratory syndrome coronavirus 2; COVID: coronavirus disease; RSV: relative search volume

Search Term	Brief Description	Average RSV	Percent of Total Inquiries (%)
SARS-CoV-2	Severe acute respiratory syndrome coronavirus 2 (the virus that causes COVID-19)	0	0
COVID19	Variant phrasing of coronavirus disease (resulting from infection with SARS-CoV-2)	0.246	0.925
COVID-19	Variant phrasing of coronavirus disease (resulting from infection with SARS-CoV-2)	1.217	4.57
Coronavirus	type of RNA virus that may cause disease	12.246	45.97
COVID	Variant phrasing of coronavirus disease (resulting from infection with SARS-CoV-2)	12.928	48.53

**Figure 4:**
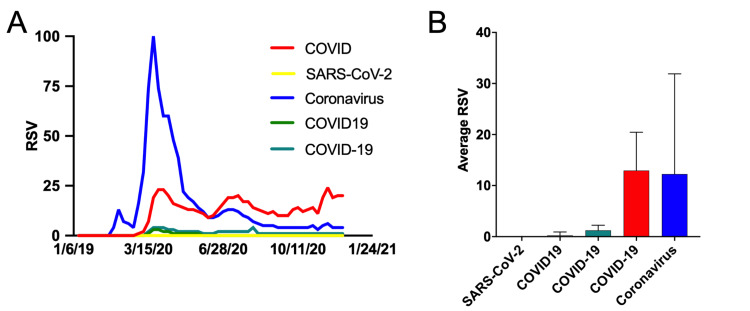
COVID-19 pandemic-related syntax selection for increased search sensitivity. (A) Relative search volume of COVID-19 pandemic-related search phrases. (B) Average relative search volume of COVID-19 pandemic-related search phrases throughout the ongoing pandemic. SARS-CoV-2: severe acute respiratory syndrome coronavirus 2; COVID: coronavirus disease; RSV: relative search volume

The temporal stacked RSV data from early December 2019 through late February 2021 was compiled to observationally determine which syntax was and is most commonly used to refer to the COVID-19 pandemic as a whole (Figure 4A). Based on the temporal data alone, the terms “COVID” and “coronavirus” appear to garner the most public interest, with coronavirus achieving the highest RSV of all search terms around mid-March 2020, then plummeting to RSV under 10 by June 2020. To control for short periods of increased interest, we calculated the average RSV of each phrase. The terms “SARS-CoV-2,” “COVID19,” and “COVID-19” had average RSV of 0, 0.25, and 1.22, respectively (Figure 3B). Together, these three terms made up roughly 5.5% of the total search queries screened. However, “COVID” and “coronavirus” had nearly the same average RSV of 12.93 ± 7.52 and 12.25 ± 19.65, respectively, and together accounted for nearly 95% of the queries screened (Figure 3B). Ultimately, we used “COVID” as the sensitivity enhancing syntax because it totaled more than 48% of the screened search inquiries related to COVID-19, and of the two significant syntaxes (“COVID” and “coronavirus”), it had the smallest standard deviation, which indicates a more consistent usage over time given the stable nature of the RSV as apparent in Figures 4A, 4B. 

Trends in COVID-19-specific search terms 

**Figure 5:**
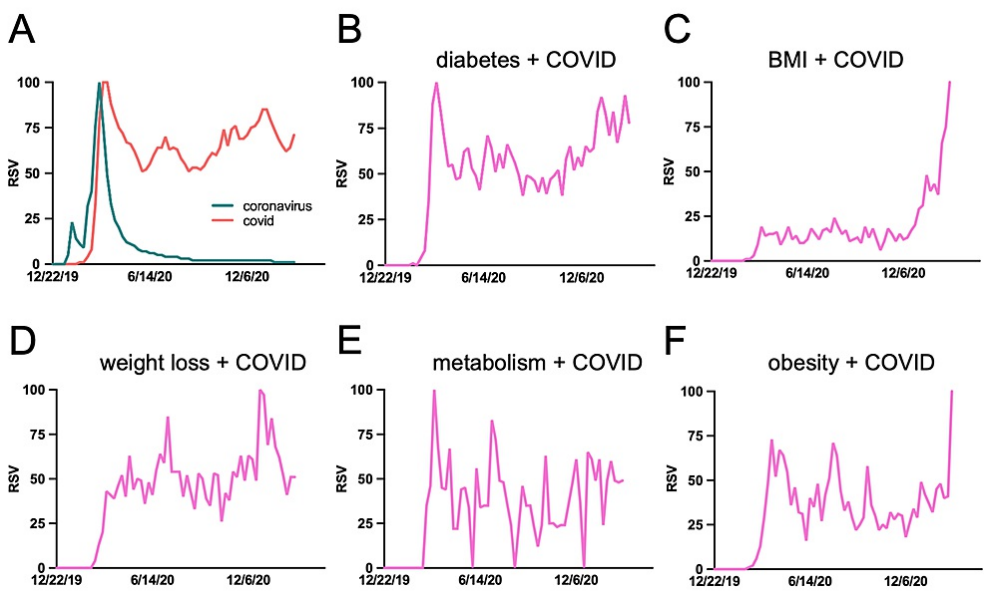
RSV of COVID-19-specific search phrases related to metabolic health during the COVID-19 pandemic (changing public interest during the pandemic progression). RSV of (A) coronavirus/COVID, (B) diabetes + COVID, (C) BMI + COVID, (D) weight loss + COVID, (E) metabolism + COVID, (F) obesity + COVID. COVID: coronavirus disease; RSV: relative search volume

After including “COVID” as the sensitivity-enhancing syntax, another round of Google Trend analysis was carried out using general terms + “COVID” in the search inquiry. The search terms focusing on metabolic health were selected for enhanced investigation. The terms “obesity + COVID,” “weight loss + COVID,” “BMI + COVID,” “diabetes + COVID,” and “metabolism + COVID” were searched in GT from February 23, 2020, to February 28, 2021, with RSV plots of “COVID” and “coronavirus” over the same time period for reference (Figures 5A-5F). Figure 5A plots RSV of “COVID” and “coronavirus” over the same time period for reference. Analyzing public interest in these search phrases during the progression of the COVID-19 pandemic requires more than computing the average RSV (Table 3).

**Table 3:** Mapping interest in metabolic-health-related search terms during the ongoing COVID-19 pandemic. COVID: coronavirus disease; RSV: relative search volume

Search Term	Date of Peak Interest	Slope of Fit Line Since Pandemic Onset	Average RSV
Obesity COVID	February 28, 2021	0.369	37.519 ± 18.113
Weight loss COVID	December 27, 2020	0.499	49.819 ± 18.517
BMI COVID	February 28, 2021	0.213	19.944 ± 17.695
Diabetes COVID	March 22, 2020	0.580	58.370 ± 18.589
Metabolism COVID	March 22, 2020	0.385	39.296 ± 21.304

**Figure 6:**
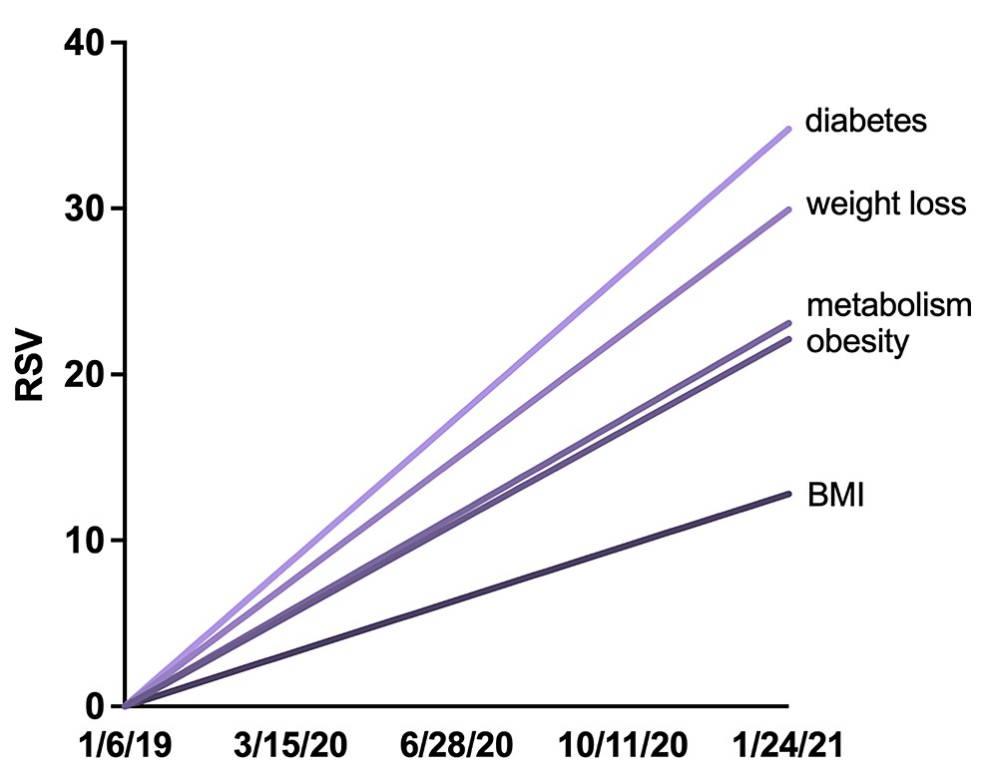
Slopes of search phrases including “COVID” since the start of the pandemic. COVID: coronavirus disease; RSV: relative search volume

To estimate the overall trend in public interest during the pandemic, the slopes of each phrase’s RSV was calculated with the assumptions that the slopes were confined by X = 0, Y = 0 and that slopes generated from linear regression would give insight into the relative trend of the search phrase (i.e., phrases with larger slopes would have increasing public interest). The slopes of “diabetes COVID,” “weight loss COVID,” “metabolism COVID,” “obesity COVID,” and “BMI COVID” were 0.58, 0.50, 0.39, 0.37, and 0.213, respectively (Figure 6 and Table 3). It is worth pointing out that fitting a trendline to the RSV may not tell the entire story. For example, though “BMI COVID” and “obesity COVID” had the lowest average RSV (19.944 ± 17.695 and 37.519 ± 18.113, respectively), their RSV saw parabolic growth since early December 2020, while “metabolism COVID” and “diabetes COVID” had RSV that plateaued after their initial spike in the public interest.

Stationarity in search phrases

In order to determine the inclusion of trend (regardless of directionality) or the deviation from a historical normal, augmented Dickey-Fuller tests were carried out to compute each search term’s ADF values (Table 4). The regression type used in the test accounts for linear trend and a constant value as our series does not have a mean = 0. The stationarity property of RSV offers us insight into whether or not trending search volume would return to "normal" or whether public interest shifts would be permanent. Many of the keywords maintained their non-stationarity through both time intervals, these include "obesity," "diet," "weight loss," and "asthma." No information on trend permanence can be gleaned from a non-stationary-to-non-stationary transition. Some keywords showed maintenance of belonging to a stationary process. The stationary-to-stationary keywords include "metabolic health," "how to improve diet," and "fitness." "Fitness" maintains the same trend in pre and post-COVID-19, while "metabolic health" transitions between stationary processes over the same time interval. These results offer insight into a growing interest in using RSV over the specified time specifically for the keyword "metabolic health." Both stationary to non-stationary and non-stationary-to-stationary represent a significant change in the permanence of trend changes. These keywords include "metabolism," "exercise," "home exercise," "health," "how to improve fitness," "how to improve health," and "BMI." Accounting for stationarity, percent change pre and post-pandemic onset, and shape of trend offers insight into which areas of metabolic health public interest is either shifting towards or away from.

**Table 4:** Dickey-Fuller test results to determine stationarity of general search terms pre and post-COVID. COVID: coronavirus disease; RSV: relative search volume

Search Term	Pre-COVID			Post-COVID		
	ADF Statistic	Stationary	p-Value	ADF Statistic	Stationary	p-Value
Coronavirus	-27.534	Yes	0.000	-6.748	No	0.000
COVID	-0.131	No	0.946	-4.107	Yes	0.001
Obesity	-2.43	No	0.134	-2.64	No	0.085
Metabolism	-0.777	No	0.826	-3.876	Yes	0.002
Metabolic health	-2.98	Yes	0.037	-5.824	Yes	0.000
Diet	-3.762	No	0.003	-2.509	No	0.113
Exercise	-3.908	Yes	0.002	-3.567	No	0.006
Home exercise	-5.798	Yes	0.000	-2.470	No	0.123
Weight loss	-3.239	No	0.018	-2.773	No	0.062
Asthma	-1.784	No	0.388	-4.51	No	0.000
Fitness	-3.507	Yes	0.008	-0.659	Yes	0.857
Health	-4.300	Yes	0.000	-2.714	No	0.072
BMI	-4.048	Yes	0.001	-1.482	No	0.542
How to improve fitness	-4.495	Yes	0.000	-3.725	No	0.004
How to improve health	-2.02	No	0.278	-3.657	Yes	0.005
How to improve diet	-8.14	Yes	0.000	-6.65	Yes	0.000

## Discussion

Google Trends offers a unique methodology to obtain useful data on how public interest in certain areas changes over time and relative to other terms. Relatedly, the public interest may be used as a suitable proxy to judge scientific communication efficiency. The COVID-19 pandemic is arguably one of the most transparent medical events in modern history, with considerable news coverage, daily alerts, and publicized scientific findings. To evaluate whether or not clinical information regarding the impact of metabolic health on COVID-19 outcome has been effectively communicated, we carried out a variety of analyses on several search phrases related to the subject. Overall, the temporal RSV corresponded to key events throughout the pandemic (i.e., a spike in comorbidities RSV following the immediate pandemic onset). Notably, the public interest in “diet” and “fitness” both significantly decreased following the onset of the COVID-19 pandemic. We hypothesize that the decreased public interest in “diet” and “fitness” may be two-folds. In one scenario, fitness and dieting are often carried out for social standards and the prolonged stay-at-home orders may have disincentivized efforts towards maintaining diets and engaging in fitness-related activities. It is also possible that the impact diets and fitness have on metabolic health is not clear to the public. The public interest in “BMI,” “weight loss,” “exercise,” and “metabolic health” trended up (with varying degrees of significance), likely as a result of two factors: (1) the unintentional weight gain during the progression of the pandemic led to increased interest in improving health overall and (2) information regarding the impact of each phrase relative to the clinical outcome was effectively communicated. It is apparent that the public search interest indicates an overall desire to improve health, though the decrease in public interest in dieting and fitness might suggest the public lacks information on how to improve their overall health. 

Throughout the progression of the pandemic, distinct increases (and decreases) in social interest in metabolic health-related topics could have been assessed for increased efforts in communicability. Google Trends is a free and extremely effective tool to gauge how effectively information is disseminated to the public. As such, it may be advantageous for clinicians and science communicators to frequently map changing public interest in phrases directly associated with ongoing health events as benchmark measurements for public interest/knowledge.

## Conclusions

The onset of the COVID-19 pandemic brought about a significant change in public and personal health policy. The long-term lockdowns, restricted access to gyms and certain foods/stores, and unprecedented job loss may all contribute to changing public interest in metabolic health-related search phrases. Additionally, weight gain during the pandemic has been cited as one of the most profound unwanted side effects of the prolonged lockdown, though the long-term implications for lockdown-associated weight gain are unknown. We identified phrases with increased, decreased, or unchanged public interest relating to metabolic health that may serve to better inform clinical dietitians, nutritionists, and other healthcare providers as to what information is being over or under communicated. While some phrases had increases in the public interest, two important terms, “diet” and “fitness” trended down significantly. Decreasing or stagnant public interest in phrases associated with improving clinical outcomes following COVID-19 may serve as indicators for where increased attention and communicability of clinicians should be focused. The application of public interest data in clinics may offer benchmarks for increasing emphasis on certain nutritional and exercise aspects relative to the ongoing pandemic.
